# Drug-Facilitated Sexual Assault Pornography and Sexual Violence While Partying: Cross-Sectional Study

**DOI:** 10.2196/80110

**Published:** 2026-01-22

**Authors:** Pablo Prego-Meleiro, Guadalupe Pastor-Moreno, Irantzu Recalde-Esnoz, Luis Sordo

**Affiliations:** 1 Department of Public Health and Maternal and Child Health Faculty of Medicine Universidad Complutense de Madrid Madrid Spain; 2 Centro de Investigación Biomédica en Red de Epidemiología y Salud Pública Madrid Spain; 3 Instituto Universitario de Investigación en Ciencias Policiales Universidad de Alcalá Alcalá de Henares Spain; 4 Observatorio Universitario de Violencia Sexual Facilitada por Drogas Universidad de Alcalá Alcalá de Henares Spain; 5 Instituto de Investigación Biosanitaria de Granada Granada Spain; 6 Andalusian School of Public Health Granada, Andalusia Spain; 7 Department of Sociology and Social Work Universidad Publica de Navarra Pamplona, Navarre Spain; 8 Fundación para la Investigación Biomédica del Hospital Clínico San Carlos (IDISCC) Madrid Spain

**Keywords:** drug-facilitated sexual assault, drugs, sexual assault, pornography consumption, sexual violence

## Abstract

**Background:**

Drug-facilitated sexual assaults (DFSAs) in youth partying contexts represent a growing public health concern, affecting approximately half of women and 1 in 4 men. These assaults often occur in environments where alcohol and other psychoactive substances are consumed, leading to impaired consent and increased vulnerability. At the same time, young people are increasingly exposed to pornography, often using it as a primary source of sexual information. However, pornography can disseminate misleading or harmful messages about sexuality and consent. Of particular concern is a subtype of pornographic material (hereafter referred to as DFSA pornography) that depicts nonconsensual sexual acts involving individuals who are asleep, unconscious, or under the influence of psychoactive substances, including alcohol and other drugs.

**Objective:**

This study aimed to examine the prevalence of DFSA pornography consumption among young adults in Spain and analyze its association with self-reported DFSA perpetration and victimization in party settings.

**Methods:**

A cross-sectional online survey (computer-assisted web interviewing) was conducted among individuals aged 18 to 35 years residing in Spain. Participants (N=1601; n=1534, 95.8% valid responses) were recruited from a certified online panel using quota sampling stratified by sex, age group, and region to ensure national representativeness. The questionnaire was adapted from the Sexual Experiences Survey–Short Form Victimization and the Spanish Macro-Survey on Violence Against Women. It assessed DFSA perpetration and victimization in partying contexts under the influence of alcohol or drugs. A specific variable, DFSA pornography, was created to measure intentional viewing of explicit sexual content depicting unconscious or intoxicated individuals. Additional sociodemographic variables included sex, age, educational level, sexual orientation, political ideology, nationality, and socioeconomic level. Descriptive, bivariate, and binary logistic regression analyses were performed, estimating associations between DFSA experiences and both general and DFSA pornography consumption.

**Results:**

Among respondents (800/1593, 50.2% female participants; mean age 27.0, SD 5.1 years), 78.4% (1233/1572) identified as heterosexual, and 52% (825/1587) held a university degree. Overall, 66.6% (1013/1521) reported consuming pornography in the previous year, with higher prevalence among male participants (638/753, 84.7%) than among female participants (370/762, 48.6%). DFSA pornography consumption was reported by 22.2% (167/753) of male participants and 11.3% (86/762) of female participants, and increased with overall pornography use frequency. Multivariate logistic regression indicated that DFSA perpetration (adjusted odds ratio 3.78, 95% CI 1.72-8.28; *P*<.001) and victimization (adjusted odds ratio 1.86, 95% CI 1.24-2.78; *P*=.003) were associated with DFSA pornography consumption.

**Conclusions:**

The findings reveal an association between exposure to DFSA pornography and both DFSA perpetration and victimization among young people in Spain. These results underscore the need for comprehensive sexual education that critically addresses pornography as a source of misinformation, emphasizing accurate understanding of consent and substance-impaired sexual activity. Public health strategies should integrate media literacy and consent education to mitigate the normalization of sexual violence depicted in pornography.

## Introduction

Sexual violence is a pervasive public health concern that demands immediate attention [1]. According to the World Health Organization, sexual violence encompasses unwanted sexual acts, comments, or insinuations, from verbal harassment to forced penetration [1,2]. Drug-facilitated sexual assault (DFSA) is a specific form of sexual violence in which perpetrators take advantage of individuals who are incapacitated by psychoactive substances, including alcohol, illicit drugs, or medications, consumed either voluntarily or involuntarily [1,3]. DFSA episodes particularly affect partying, dating, and hookup contexts, where alcohol and other drug use is often combined with high expectations for sexual interaction [3,4]. This type of violence occurs across diverse settings worldwide, including Europe, within which Spain has shown particular concerns [5]. A recent study showed that 1 in 2 women and 1 in 4 men among young people in Spain have experienced DFSA at least once while partying [6]. The relationship between nightlife partying and DFSA has been a focus of intense research in Spain in recent years [7-11]. Multiple studies have highlighted how rape culture leads young people of both sexes to normalize sexually violent practices such as nonconsensual sex, thereby rendering sexual violence invisible and minimizing its impact [12-16].

Pornography consumption is widespread among the young population, especially men, with prevalence of consumption in the last year ranging between 60% and 80% in Spain and other high-income countries [17-21]. In addition, this consumption has significantly increased in recent years, driven by its growing accessibility online [19,21,22]. One of the main problems related to the consumption of pornography is that many young people perceive it as a source of sexual education, turning to it for information in the absence of reliable and quality guidance; in Spain, just over 10% of young people express satisfaction with the sexual education they have received, whereas approximately half report that pornography helps them better understand and learn about sex and acknowledge using it as a source of inspiration [19]. Similar figures have been observed in other countries [23], highlighting how particularly young men incorporate practices depicted in pornography into their sexual lives [24,25].

Pornography can be a source of misinformation about sex [26,27]. Violent content in pornography can encompass a wide range of physical, verbal, and sexual violence. Numerous studies have highlighted that mainstream pornography often portrays violent scenes featuring practices characterized by aggression that demean and sexually objectify people [23,28,29]. This type of pornographic content also includes scenes of nonconsensual sexual activity while someone is asleep, unconscious, or under the influence of alcohol or other drugs [29,30]. This pornographic content resembles DFSA situations, where individuals are rendered incapacitated by the psychoactive effects of licit or illicit substances, including alcohol, other drugs of abuse, and medications. There are no data available on the consumption of this type of pornography with DFSA content. Nevertheless, as a proxy, in Spain, at least 18.2% of men and 14.5% of women among young people admit to consuming pornography classified as highly violent, particularly degrading, or humiliating [18]. These are particularly concerning figures considering that numerous studies indicate that some young people do not distinguish between pornography and real-life sexual behaviors, potentially leading to the normalization of degrading acts or extreme violence [31]; 31% of young people in Spain agree that pornography shapes sexual fantasies involving either the perpetration or reception of violence, which they perceive as a harmful effect of pornography on its consumers [18]. Therefore, given the high prevalence of DFSA in party contexts, combined with the widespread use of pornography as a sexual education source and the accessibility of violent content, this study aimed to examine the prevalence of DFSA pornography consumption and its relationship with having either experienced or perpetrated DFSA while partying.

## Methods

### Design and Participants

This was a cross-sectional study using an anonymized, self-administered online survey for sexual victimization and perpetration (computer-assisted web interviewing). The study population consisted of individuals aged 18 to 35 years residing in Spain, totaling 9,250,779 people in 2022, with 50.85% being men [32]. A minimum sample size of 1537 individuals was required, assuming 50% prevalence, 95% confidence, and a 2.5% margin of error, with a prevalence of 0.5 due to the lack of prior data as recommended by statistical guidelines [33]. Participants were recruited from an online panel managed by a research company certified under ISO 20252, which provides verified samples for social and health research. The panel includes more than 100,000 registered members residing in Spain. Panelists are recruited through paid social strategies and undergo a validation process, with sociodemographic profiles periodically updated to ensure data accuracy.

Quota sampling was applied to ensure proportional representation by sex, age group (18-24 and 25-35 years), and region (17 autonomous communities) according to the demographic distribution of the Spanish population. Study participants were selected through a routing system designed to minimize self-selection bias and received personalized invitations via email and mobile notifications. To maintain quota compliance, invitations were dynamically adjusted based on response and dropout rates.

Survey access included duplicate checks, captcha verification, and validation of self-reported gender and age. Standard quality controls, such as attention-check questions, a minimum completion time threshold, and mandatory responses, were applied to ensure data integrity.

Of the 1707 invited individuals, 106 (6.2%) declined participation or did not complete the questionnaire, resulting in a final sample of 1601 respondents.

The questionnaire was specifically tailored by an interdisciplinary team, drawing on sources such as the Sexual Experiences Survey–Short Form Victimization [34] and the 2019 Macro-Survey on Violence Against Women [35]. At the time this study was conducted, to the authors’ knowledge, no fully validated instruments existed to specifically assess DFSA perpetration and victimization or the use of pornography depicting DFSA content. A pretest assessed clarity, reliability, and validity, including cognitive debriefing with over 100 participants to address any understanding issues and ensure clarity. Questions were designed to avoid identifiable information and social desirability biases. IP addresses and cookies were not collected.

### Variables

Two main variables provided information about DFSA perpetration or victimization experiences while partying. Sexual violence included unwanted sexual touching, kissing, masturbation by another person, oral sex, or penetration. DFSA perpetration was defined as “having engaged in any of these sexual behaviors non-consensually with someone who was under the influence of alcohol or other drugs and, because of that, was unable to communicate their sexual consent, while partying or immediately afterwards” (“no” or “yes”). DFSA victimization was defined as “having experienced any of these sexual acts non-consensually when you were under the influence of alcohol or other drugs, while partying or immediately afterwards” (“no” or “yes”).

Variables concerning pornography use were pornography consumption frequency, defined as “how often one intentionally seeks out and views videos or photographs containing explicit sexual content” (“no consumption,” “Yes, between every day and two to three times per week,” “Yes, between once per week and two to three times per month,” and “Yes, less than once per month”) and DFSA pornography consumption, defined as “seeking out and intentionally viewing videos or photographs containing explicit sexual content in which a person is asleep, unconscious, or under the influence of alcohol or other drugs (including sedation or other effects)” (“no” or “yes”). The item assessing DFSA pornography consumption was developed and reviewed by the research team to ensure conceptual clarity. During the pilot questionnaire, all participants completed the item, and only 1 reported discomfort, indicating good comprehension and acceptability. Other independent variables were sex; age; educational level (university or nonuniversity); sexual orientation (heterosexual or nonheterosexual); political ideology (left or right), originally measured on a scale from 1 to 10 and dichotomized into 1 to 5 for “left” and 6 to 10 for “right”; nationality (Spanish or Spanish and/or other); and socioeconomic level (family income [net per month in €]; low and low to medium: ≤€2000 [US $2357.44]; medium to high or high: >€2000 [US $2357.44]). Details on variable measurement and the full list of items are provided in the questionnaire in Multimedia Appendix 1.

### Statistical Analysis

A descriptive analysis of pornography use focusing on the overall and gender-specific prevalence rates of pornography consumption frequency and DFSA pornography consumption was conducted. Relationships between the 2 main variables (DFSA perpetration and DFSA victimization) and covariates were examined using the chi-square and Fisher exact tests for categorical variables and ANOVA for continuous variables. Binary logistic regression models were performed to explore the relationship between DFSA perpetration and DFSA victimization and pornography use including all sociodemographic variables, with *P*<.05 in the bivariate analysis. Odds ratios and their corresponding 95% CIs were provided. Data analyses were conducted using Stata (version 19; StataCorp).

### Ethical Considerations

Ethics approval was granted by the Research Ethics Committee of the University of Alcalá (approval code CEIP2022/2/040) on March 31, 2022. Informed consent was obtained from all participants before taking part. Participation was voluntary, and participants were free to decline or withdraw at any time. Privacy and confidentiality were ensured through the use of an anonymous survey. No personal identifiers were collected, and all data were analyzed in aggregate form. Consequently, individual participants cannot be identified from the dataset.

As a result of their participation in the surveys, panelists receive incentive points that can be redeemed through a catalog of gifts, prize draws, and even donations to nongovernmental organizations. These incentives are flexible and tailored to the specific conditions of each survey, taking into account factors such as survey duration, and are designed to reward panelists according to their level of commitment and effort. The incentive system allows for variations to be defined according to the survey process, ensuring that panelists receive appropriate compensation for their time and contributions. This approach not only motivates panelists to participate actively in surveys but also encourages them to provide thoughtful and comprehensive responses, resulting in higher-quality data by optimizing participant engagement and maximizing response rates.

## Results

Among the 1601 respondents, (800/1593, 50.2%) were female participants, the mean age was 27.0 (SD 5.1) years, 78.4% (1233/1572) were heterosexual, 51.9% (825/1587) had a university degree, 54.6% (689/1262) had a low to medium or low socioeconomic status, 91.4% (1446/1583) were Spanish, and 75.8% (1213/1601) had a left-wing political ideology. Ages ranged from 18 to 35 years for both male and female participants (Table 1).

**Figure 1:**
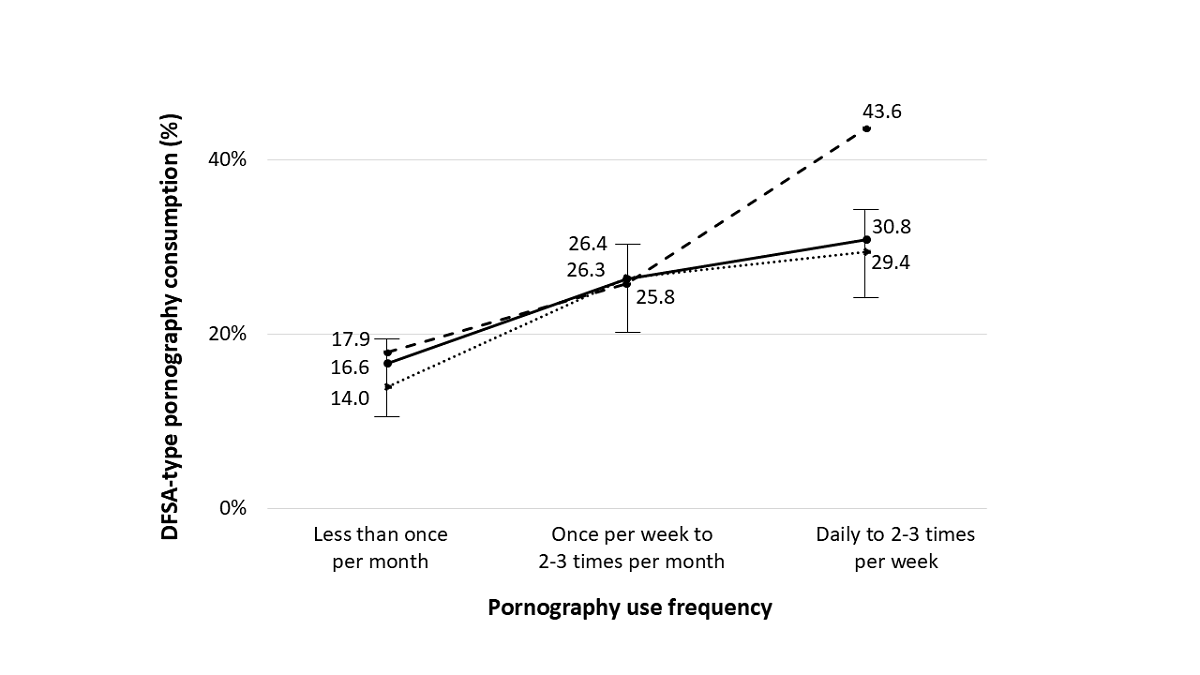
Relationship between drug-facilitated sexual assault (DFSA)–type pornography consumption and pornography use frequency (low, medium, and high), overall and by gender. The solid line represents the overall group, the dotted line represents men, and the dashed line represents women. The 95% CIs are shown for the overall group (*P*<.001).

The prevalence of pornography consumption in the last year was 66.6% (1013/1521), with 24.5% (373/1521) consuming it daily or 2 to 3 times a week. Among male participants, 84.7% (638/753) had consumed pornography, with 44.2% (333/753) consuming it daily or 2 to 3 times a week and 28.2% (212/753) consuming it between once a week and 2 to 3 times a month. Among female participants, 27.2% (207/762) reported consuming pornography less than once a month, whereas 16.3% (124/762) consumed it between once a week and 2 to 3 times a month. Regarding the type of pornography consumed, 22.2% (167/753) of male participants and 11.3% (86/762) of female participants reported consuming pornography with DFSA content (Table 2). The higher the frequency of pornography use, the greater the consumption of DFSA pornography. A total of 30.8% (115/373) of those who consumed pornography more frequently acknowledged having seen DFSA pornography (Figure 1).

The results of the bivariate analysis showed that the odds of having perpetrated DFSA at least once while partying were 6.27 times higher among those who consumed DFSA pornography (*P*<.001), whereas they were 3.01 times higher among those who had more frequent pornography consumption (daily or 2-3 times a week; *P*<.001). However, the results of the multivariate analysis model, adjusted for both variables and other sociodemographic characteristics, revealed that perpetrating DFSA while partying appeared only in relation to the type of pornography consumed, not its frequency. The odds of having perpetrated DFSA while partying were 3.78 times higher among people who reported consuming DFSA pornography than among those who did not (*P*<.001). Regarding other factors, in the multivariate analysis, DFSA perpetration was higher among male participants (adjusted odds ratio [aOR] 2.03, 95% CI 1.17-3.51; *P*=.01), nonheterosexual individuals (aOR 2.10, 95% CI 1.33-3.32; *P*<.001), and individuals of foreign origin (aOR 2.55, 95% CI 1.40-4.62; *P*=.002). The logistic model (*χ*^2^_8_=88.8; *P*<.001; McFadden pseudo-*R*^2^=0.123) fit the data well (Hosmer-Lemeshow *χ*^2^_8_=7.5; *P*=.48; Table 3).

**Table 1:** Sociodemographic characteristics of the studied sample, overall and stratified by sex (N=1601).^a^

		Global	Female (n=800)	Male (n=793)
Age (y), mean (SD; 95% CI)		27.0 (5.1; 26.8-27.3)	26.1 (4.9; 25.7-26.4)	28.0 (5.2; 27.7-28.4)
**Sexual orientation, n (%; 95% CI)**				
	Heterosexual	1233 (78.4; 76.3-80.4)	623 (79.6; 76.6-82.2)	608 (77.8; 74.7-80.5)
	Nonheterosexual	339 (21.6; 19.6-23.7)	160 (20.4; 17.8-23.4)	174 (22.3; 19.5-25.3)
**Political ideology, n (%; 95% CI)**				
	Left	1231 (75.8; 73.6-77.8)	635 (79.4; 76.4-82.0)	571 (72.0; 68.8-75.0)
	Right	388 (24.2; 22.2-26.4)	165 (20.6; 18.0-23.6)	222 (28.0; 25.0-32.1)
**Nationality, n (%; 95% CI)**				
	Spanish	1446 (91.4; 89.9-92.6)	719 (90.9; 88.7-92.7)	723 (92.2; 90.1-93.9)
	Spanish and/or other	137 (8.7; 7.4-10.1)	72 (9.1; 7.3-11-3)	61 (7.8; 6.1-9.9)
**Socioeconomic level, n (%; 95% CI)**				
	Low to medium low	689 (54.6; 51.8-57.3)	358 (57.9; 54.0-61.8)	329 (51.3; 47.4-55.2)
	Medium high to high	573 (45.4; 42.7-48.2)	260 (42.1; 38.2-46.0)	312 (48.7; 44.8-52.6)
**Educational level, n (%; 95% CI)**				
	University	825 (52.0; 49.5-54.4)	406 (51.2; 47.7-54.7)	414 (52.7; 49.2-56.1)
	Nonuniversity	762 (48.0; 45.6-50.5)	387 (48.8; 45.3-52.3)	372 (47.3; 43.9-50.8)

^a^Totals do not equal 1601 because 8 (0.5%) participants preferred not to specify their sex.

**Table 2:** Prevalence of pornography consumption categorized by use frequency and type of pornography consumed based on whether drug-facilitated sexual assault (DFSA) pornography was used (N=1521).

			Global, n (%; 95% CI)	Female, n (%; 95% CI)		Male, n (%; 95% CI)
**Total responding “yes”**			1013 (66.6; 64.2-68.9)	370 (48.6; 45.0-52.1)		638 (84.7; 82.0-87.1)
	Daily to 2 to 3 times per week	373 (24.5; 22.4-26.8)		39 (5.1; 3.8-6.9)	333 (44.2; 40.7-47.8)	
	Once per week to 2 to 3 times per month	339 (22.3; 20.3-24.5)		124 (16.3; 13.8-19.1)	212 (28.2; 25.1-31.5)	
	Less than once per month	301 (19.8; 17.9-21.9)		207 (27.2; 47.9-55.0)	93 (12.4; 10.2-14.9)	
	Pornography without DFSA	759 (49.9; 47.4-52.4)		284 (37.3; 33.9-40.8)	471 (62.6; 59.0-65.9)	
	Pornography with DFSA	254 (16.7; 14.9-18.7)		86 (11.3; 9.2-13.7)	167 (22.2; 19.3-25.3)	
No pornography use			508 (33.4; 31.1-35.8)	392 (51.4; 47.8-55.0)		115 (15.3; 12.9-18.0)

**Table 3:** The relationship between lifetime drug-facilitated sexual assault (DFSA) perpetration while partying and the type of pornography consumed and frequency of consumption (N=1482).^a^

Sociodemographic and behavioral variables			Perpetrated DFSA at any point in their life while partying					*P* value			Crude OR^b^ (95% CI)			Adjusted OR (95% CI)
			Yes		No									
**Type of pornography consumed, n (%)**								<.001						
	No consumption	18 (3.6)		485 (96.4)					Reference			Reference		
	Without DFSA	35 (4.6)		719 (95.4)					1.31 (0.73-2.34)			0.91 (0.43-1.95)		
	With DFSA	47 (18.9)		202 (81.1)					6.27 (3.55-11.06)^c^			3.78 (1.72-8.28)^d^		
**Frequency of pornography consumption, n (%)**								<.001						
	Never to less than once per month	33 (4.1)		770 (95.9)					Reference			Reference		
	Once per week to 2 to 3 times per month	25 (7.5)		310 (92.5)					1.88 (1.10-3.22)^d^			1.01 (0.49-2.06)		
	Daily to 2 to 3 times per week	42 (11.4)		326 (88.6)					3.01 (1.87-4.83)^c^			1.30 (0.65-2.59)		
**Sex, n (%)**								<.001						
	Female	32 (4.1)		757 (95.9)					Reference			Reference		
	Male	72 (9.2)		711 (90.8)					2.40 (1.56-3.68)^c^			2.03 (1.17-3.51)^d^		
Age (y), mean (SD)			27 (5)		27 (5)		.79			0.99 (0.96-1.03)			1.00 (0.98-1.05)	
**Sexual orientation, n (%)**								<.001						
	Heterosexual	61 (5.0)		1162 (95.0)					Reference			Reference		
	Nonheterosexual	42 (12.6)		292 (87.4)					2.74 (1.81-4.14)^c^			2.10 (1.33-3.32)^d^		
**Nationality, n (%)**								<.001						
	Spanish	81 (5.7)		1351 (94.3)					Reference			Reference		
	Spanish and/or other	20 (14.8)		115 (85.2)					2.90 (1.72-4.90)^c^			2.55 (1.40-4.62)^d^		
**Educational level, n (%)**								.68						
	University	56 (6.9)		759 (93.1)					Reference			—^e^		
	Nonuniversity	48 (6.4)		707 (93.6)					0.92 (0.62-1.37)			—		

^a^The multivariate model included as covariates those variables that showed a *P* value of <.05 in the bivariate analysis.

^b^OR: odds ratio.

^c^*P*<.001.

^d^*P*<.05.

^e^Variables not included in the multivariate model according to the results of the bivariate analysis.

Regarding DFSA victimization while partying, the results of the bivariate analysis were largely consistent with those of the multivariate analysis. In this model, the probability was 1.86 times higher among DFSA pornography users (95% CI 1.24-2.78, *P*=.003). This association was also 3.36 (95% CI 2.53-4.46) times higher among female participants (*P*<.001), 1.67 (95% CI 1.28-2.20) times higher among nonheterosexual individuals (*P*<.001), 1.65 (95% CI 1.10-2.47) times higher among individuals of foreign origin (*P*=.01), and 1.36 (95% CI 1.09-1.71) times higher among those with lower educational levels (*P*=.008). The overall model (*χ*^2^_9_=128.4; *P*<.001) explained 6.6% of the variance (McFadden pseudo-*R*^2^=0.066) and showed good fit to the data (Hosmer-Lemeshow *χ*^2^_8_=4.5; *P*=.81; Table 4).

**Table 4:** The relationship between lifetime drug-facilitated sexual assault (DFSA) victimization while partying and the type of pornography consumed and frequency of consumption (N=1470).^a^

Sociodemographic and behavioral variables		Experienced DFSA at any point in their life while partying		*P* value		Crude OR^b^ (95% CI)		Adjusted OR (95% CI)	
		Yes	No						
**Type of pornography consumed, n (%)**					.07				
	No consumption	190 (37.8)	313 (62.2)			Reference		Reference	
	Without DFSA	275 (36.5)	478 (63.5)			0.95 (0.75-1.20)		1.26 (0.92-1.72)	
	With DFSA	109 (44.7)	135 (55.3)			1.33 (0.98-1.81)		1.86 (1.24-2.78)^c^	
**Frequency of pornography consumption, n (%)**					.02				
	Never to less than once per month	319 (39.9)	481 (60.1)			Reference		Reference	
	Once per week to 2 to 3 times per month	137 (41.4)	194 (58.6)			1.06 (0.82-1.38)		1.28 (0.90-1.82)	
	Daily to 2 to 3 times per week	118 (32.0)	251 (68.0)			0.71 (0.55-0.92)		1.12 (0.77-1.64)	
**Sex, n (%)**					<.001				
	Male	210 (27.0)	567 (73.0)			Reference		Reference	
	Female	381 (48.4)	406 (51.6)			2.53 (2.05-3.13)^d^		3.36 (2.53-4.46)^d^	
Age (y), median (SD)		27 (5)	27 (5)	.88		1.00 (0.98-1.02)		1.03 (1.00-1.05)^c^	
**Sexual orientation, n (%)**					<.001				
	Heterosexual	430 (35.3)	790 (64.8)			Reference		Reference	
	Nonheterosexual	156 (47.7)	171 (52.3)			1.68 (1.31-2.15)^d^		1.67 (1.28-2.20)^d^	
**Nationality, n (%)**					.002				
	Spanish	521 (36.5)	905 (63.5)			Reference		Reference	
	Spanish and/or other	66 (50.0)	66 (50.0)			1.74 (1.21-2.48)^c^		1.65 (1.10-2.47)^c^	
**Educational level, n (%)**					.001				
	University	274 (34.0)	532 (66.0)			Reference		Reference	
	Nonuniversity	319 (42.2)	437 (57.8)			1.42 (1.15-1.74)^c^		1.36 (1.09-1.71)^c^	

^a^The multivariate model included as covariates those variables that showed a *P* value of <.05 in the bivariate analysis.

^b^OR: odds ratio.

^c^*P*<.05.

^d^*P*<.001.

## Discussion

This study confirms the widespread use of pornography among young people, indicating that those who use it more frequently are also more likely to consume sexually violent pornography. Additionally, evidence was provided regarding the relationship between the use of sexually violent pornography and both DFSA perpetration and victimization.

In this study, 2 out of 3 young people (1013/1521, 66.6%) admitted to consuming pornography, with consumption being almost twice as high in male participants compared to female participants. These results align with a recent nationwide study in Spain, according to which 6 out of 10 young people aged 16 to 29 years admitted to using pornography, with 72.1% being men and 59.3% being women [19]. Similarly, other studies have found that approximately 70% of men and 30% of women use pornography in high-income countries [17,18]. Regarding frequency, in our study, almost half (333/753, 44.2%) of all male participants consumed pornography daily or 2 to 3 times a week. Among female participants, consumption was much less frequent. These figures are slightly higher than those reported in similar studies conducted in Spain [19] and Europe [36]. The data from our study are more up-to-date and are based on more representative samples. Therefore, they may reflect current consumption, especially considering that pornography consumption has been growing in recent years [22,37].

In terms of type of pornography, more than 20% of male participants (167/753, 22.2%) and 10% of female participants (86/762, 11.3%) in our study reported consumption of pornography featuring scenes in which a person is asleep, unconscious, or under the influence of alcohol or other drugs (referred to as DFSA pornography). These figures cannot be directly compared as there is currently no scientific literature providing prevalence data on the consumption of violent pornographic content specifically depicting DFSA situations. As such, comparisons could be made with broader data on the consumption of violent pornography. Some studies indicate that 10% of adolescents have been exposed to violent pornography, with a gradual increase in violent themes as age progresses [38]. In this regard, a study in New Zealand highlighted how easily young people can be exposed to nonconsensual sexual behavior in online pornography, including scenes of sexual activity while someone is sleeping [39], findings similar to those described by other authors [30,31]. In Spain, 40.2% of individuals have viewed pornographic content classified as high in violence, particularly degrading, or humiliating, and 16.6% acknowledge doing so with high or moderate frequency (18.2% of men and 14.5% of women) [19]. Additionally, 5.2% of men and 6.9% of women indicate that the presence of violence is the factor that influences them the most when selecting pornographic material [19]. These results show the magnitude of pornography consumption of this type. There are many related considerations, but these are beyond the scope of this study. What we must emphasize again is that many young people acquire their sexual education through pornography (in the absence of other forms of education) [19,40], with a significant portion of this pornography being of a violent nature.

According to our study, using DFSA pornography is related to approximately fourfold higher odds of perpetrated DFSA while partying. Similarly, other researchers have observed a connection between sexual assault perpetration and the use of violent pornography [23,41]. Models explaining this relationship suggest that the risk of perpetration correlates with male pornography users who have high levels of hostility and sexual promiscuity [42]. Exposure to violent pornography also shapes sexual behavior by reinforcing scripts that are perceived as normative, acceptable, and gratifying, which are then activated and applied in dating and sexual relationships [43]. This correlation was obtained in analyses adjusting for other factors. In addition to the type of pornography consumption, it was noted in this study that there were more perpetrators among male participants, foreigners, and nonheterosexual individuals. The presence of men in these studies is nothing new [23,44], hence the importance of adjusting the results by gender. Regarding country of origin and sexual orientation, these categories encompass highly diverse populations. These groups are heterogeneous and shaped by intersecting social determinants that may influence risk and experiences in complex ways [45]. Further research is needed stratifying by country of origin and other relevant factors [46,47].

Concerning victimization, experiencing DFSA while partying was twice as frequent among users of DFSA pornography content. Although various studies have indicated a link between sexual victimization and exposure to pornography [44,48,49], there is currently no known research specifically addressing DFSA. On the one hand, many studies on violence against women suggest that conventional pornography often depicts violent scenes that sexually degrade and objectify women [23,28,29,40]. It is noted that female sexual objectification in pornography is linked to a higher likelihood of sexual victimization in young women due to the normalization of or desensitization to violent sexual behaviors [50]. On the other hand, studies linking violence using psychoactive substances and pornography primarily focus on alcohol, consistently finding an increased risk of sexual victimization among women who consume pornography [48]. While it is difficult to infer a cause, it has been suggested that increased exposure to violent pornography and its normalization, combined with the effects of alcohol, may impair the initial ability to detect an aggression [51]. At the same time, considering the absence of temporality in cross-sectional designs, the observed relationship between having experienced DFSA and DFSA pornography consumption leads us to hypothesize that, in the absence of adequate social support, some DFSA survivors may resort to viewing such content to understand the episode even at the risk of revictimization. However, this hypothesis is not supported by empirical data, suggesting directions for future research. Regardless, this highlights the imperative to strengthen support systems for survivors of DFSA. Consequently, it is essential to conduct longitudinal studies that establish causal relationships and qualitative research that explores DFSA survivors’ experiences in depth. In the same way, related adjustment variables indicate that this is an issue in which global messages must be accompanied by other messages particularized according to gender, educational level, or country of origin.

Considering the high prevalence of DFSA experiences in youth party settings [6] and the fact that many young people turn to pornography as a source of sexual education [19], the influence of DFSA-themed pornography in normalizing this form of sexual violence among young people is deeply concerning. While we cannot establish a causal relationship, there is a clear correlation between DFSA—whether perpetration or victimization—and the consumption of this specific type of pornography. This correlation does not emerge when considering the overall quantity of pornography consumed. Although higher levels of consumption are related to the consumption of violent porn, it is specifically this violent content that shows a significant correlation with DFSA. Consequently, we should not wait for causal evidence to implement the necessary sexual health promotion interventions. Preventive action is essential, particularly to ensure that the normalization of sexual behaviors is not shaped primarily by the content of the pornography that young people consume.

This study’s findings contribute to addressing some global challenges highlighted by the United Nations Sustainable Development Goals, particularly those aimed at ending violence and fostering just societies. As DFSA is a paradigmatic example of how violence affects the most vulnerable individuals, this study reinforces the commitment to “leave no one behind” in achieving sustainability.

This study has several limitations. First, the cross-sectional design precludes causal inference. Second, although quota sampling approximated the demographic distribution of the Spanish population in terms of age, gender, and region, participation was voluntary and based on an online panel, which may have introduced self-selection bias and limited generalizability. Social desirability bias cannot be entirely ruled out despite assurances of anonymity. In addition, the representation of sexually diverse and gender-diverse groups was limited, and the sample size did not allow for analyses by DFSA subtype or level of invasiveness. Third, although the questionnaire was piloted, some constructs—such as the use of DFSA-related pornographic content—were assessed using nonvalidated items, which may affect measurement precision. Finally, no psychological support was available for participants who might have experienced discomfort. Despite these limitations, this study provides novel and valuable evidence on DFSA-related pornographic content and its association with sexual violence perpetration and victimization in party contexts.

The use of pornography depicting nonconsensual sex involving individuals who are asleep, unconscious, or under the influence of alcohol or other drugs correlates with experiences of DFSA perpetration and victimization in youth party settings. This association emphasizes the importance of addressing the impact of pornography as a primary source of sexual knowledge for young people, particularly in a context in which the widespread use of pornography intersects with insufficient sexual education and a lack of effective mechanisms to control consumption among minors. Future public health strategies may include ongoing research regarding the implications of pornography for sexual violence. From a public health policy perspective, it is recommended to provide training programs for educators and clinicians to enhance their skills in sex education considering the realities of pornography and its influence on young people. Accurate information on these realities should also be integrated into comprehensive sexual education initiatives and educational materials aimed directly at youth. Finally, legal responsibilities should be expanded, and the obligations of online pornography distribution platforms should be strengthened to improve the monitoring, filtering, and removal of violent content.

## Appendix

Multimedia Appendix 1 Questionnaire used in the study.

## Abbreviations

aOR: adjusted odds ratio
DFSA: drug-facilitated sexual assault

## Data Availability

The datasets generated or analyzed during this study are available from the corresponding author on reasonable request.
