# Supplementary material for: Drug-Facilitated Sexual Assault Pornography and Sexual Violence While Partying: Cross-Sectional Study
Source: JMIR Public Health Surveill. 2026 Jan 22;12:e80110. doi: 10.2196/80110 (PMC12877744; doi:10.2196/80110)
Supplement: Multimedia Appendix 1 [file publichealth_v12i1e80110_app1.docx]

**Appendix 1: Questionnaire (English Translation)**

*The questionnaire used was more extensive. Only the set of questions that generated the variables used for this study is shown.*

**Participation Note:** Participation is completely voluntary and anonymous. All shared data are subject to the Spanish Organic Law 3/2018, of December 5, on Personal Data Protection and Guarantee of Digital Rights. We guarantee the absolute anonymity and confidentiality of your responses, in strict compliance with the laws on statistical secrecy and personal data protection.

It is very important that you answer honestly. Please remember that everything you say is completely confidential. Once the information is recorded anonymously, individual questionnaires are destroyed.

Some questions address intimate aspects of sexual life and experiences, so we thank you in advance for your honest answers. Thank you for participating in our survey.

**Do you agree to participate in this survey?**

- Yes
- No

**Next, we will ask you about your sexual experiences in the past. It is very important that you answer honestly. Everything you say is completely anonymous and confidential.**

**Have you ever in your life, while partying or immediately after, experienced any of the following episodes without your consent while under the influence of alcohol or other drugs?**

| **Behavior** | **Yes** | **No** | **Prefer not to answer** |
| --- | --- | --- | --- |
| Kissing |  |  |  |
| Touching |  |  |  |
| Masturbation |  |  |  |
| Oral sex (fellatio, cunnilingus) |  |  |  |
| Vaginal penetration |  |  |  |
| Anal penetration |  |  |  |

**Do you think that, at any time while partying or immediately after, you have engaged in any of these behaviors toward someone who was under the influence of alcohol or other substances and could not communicate their sexual consent?**

| **Behavior** | **Yes** | **No** | **Prefer not to answer** |
| --- | --- | --- | --- |
| Kissing |  |  |  |
| Touching |  |  |  |
| Masturbation |  |  |  |
| Oral sex (fellatio, cunnilingus) |  |  |  |
| Vaginal penetration |  |  |  |
| Anal penetration |  |  |  |

**Indicate your sex as it appears on your ID card, passport, or a similar official document:**

- Male
- Female
- Prefer not to answer

**How old were you on your last birthday? Enter your age in digits:**

**From the following list, which option best describes your sexual orientation?** By sexual orientation, we mean which sex you are attracted to.

- Heterosexual (people of the opposite sex)
- Homosexual (people of your same sex)
- Bisexual (people of both sexes)
- Other sexual orientation
- I haven’t decided yet / I don’t know
- Prefer not to answer

**How often do you watch pornography?** By watching pornography, we mean intentionally searching for and viewing videos or photographs with explicit sexual content.

- Every day or almost every day (4 times or more per week)
- 2 or 3 times per week
- Once a week
- 2 or 3 times per month
- Less than once a month
- I never watch pornography
- Prefer not to answer

**When you watch pornography, on a scale from 1 to 10, where 1 is never and 10 is always, how often do you see scenes in which a person is asleep, unconscious, or under the influence of drugs (sedation or other effects)?**

**When discussing politics, the terms left and right are commonly used. On a scale from 1 to 10, where 1 means "far left" and 10 means "far right," where would you place yourself on this scale?**

**Regarding nationality, your nationality is:**

- Spanish
- Spanish and another nationality
- Another nationality
- Prefer not to answer

**Currently, among all the members of your household living with you, including yourself, approximately, what is your total monthly net income?**

- No monthly income
- Less than or equal to €1,000
- Between €1,001 and €2,000
- Between €2,001 and €3,000
- Between €3,001 and €4,000
- More than €4,000
- I don’t know
- Prefer not to answer

**What is the highest level of education you have completed?**

1. Less than primary education
2. Primary education
3. Secondary education
4. Basic vocational training
5. Intermediate vocational training
6. High school / Baccalaureate
7. Higher vocational training
8. University studies (Bachelor’s degree, Licentiate, etc.)
9. Postgraduate studies (Master’s, Postgraduate, Doctorate, etc.)
10. Prefer not to answer
